# High-risk Human Papillomavirus Testing for Triage of Women with Previous Cytological Abnormalities from the Vale do Ribeira Region

**DOI:** 10.1055/s-0040-1712992

**Published:** 2020-06

**Authors:** Sandra Lorente, Natália Coelho Couto de Azevedo Fernandes, Daniela Etlinger-Colonelli, Rodrigo Albergaria Réssio, Sonia Maria Pereira de Oliveira, Regina Maria Catarino

**Affiliations:** 1 Departamente of Pathological Anatomy, Instituto Adolfo Lutz, São Paulo, SP, Brazil

**Keywords:** human papillomavirus, screening, cervical cancer, papanicolaou test, papilomavírus humano, rastreamento, câncer do colo do útero, papanicolaou

## Abstract

**Objective** To evaluate the performance of the hybrid capture 2 (HC2) high-risk papillomavirus (hrHPV) assay and cytological test in women with previous abnormalities, to detect cervical intraepithelial neoplasia grade 2 or worse (≥ CIN 2).

**Methods** A cytological test and HC2 (Qiagen, Gaithersburg, Maryland, EUA) for hrHPV were conducted in 359 liquid-based (Sure Path, Becton Dickinson, TriPath Imaging, Burlington, NC, USA) samples collected from women from the Vale do Ribeira Region, during July 2013 and September 2015 with previous cytology classified as atypical squamous cells of undetermined significance (ASC-US), low-grade squamous intraepithelial lesion (LSIL), atypical squamous cells, cannot exclude high-grade squamous intraepithelial lesions (ASC-H), and atypical glandular cells (AGC). The histopathological examination was conducted in 179 women. The performance evaluations were calculated using the “exact” Clopper-Pearson 95% confidence interval (CI) test by MEDCALC (Medcalc Software Ltd, Ostend, Belgium).

**Results** The ≥ CIN 2 frequency was 11.7% (21/179). The HC2 for hrHPV and repeat cytology to detect ≥ CIN 2 obtained, respectively, a sensitivity of 90.5% (95%CI = 69.6–98.8) and 90.5%, (95%CI = 69.6–98.8), a specificity of 65.8% (95% CI = 57.9–73.2) and 43.7% (95%CI = 35.8–51.8), a positive predictive value of 26.0% (95% CI = 21.4–31.3) and 17.6%, (95%CI = 14.9–20.6), and a negative predictive value of 98.1% (95%CI = 93.3–99.5) and 97.2% (95% CI = 90.1–99.2).

**Conclusion** Hybrid capture 2 for hrHPV improves the performance of the detection of ≥ CIN 2, without compromising sensitivity, and provides a greater safety margin to return to the triennial screening of women undergoing follow-up due to previous abnormalities, without underlying ≥ CIN 2.

## Resumo

**Objetivo** Avaliar o desempenho da captura híbrida 2 (CH2) para papilomavírus humano de alto risco (HPVar) e repetição do exame citopatológico em mulheres com anormalidades em citologia anterior, para detectar neoplasia intraepitelial cervical grau 2 ou pior (≥ NIC 2).

**Métodos** Foi realizado exame citopatológico e CH2 para HPVar (Qiagen, Gaithersburg, Maryland, EUA) em 359 amostras em meio líquido (Sure Path, Becton Dickinson, TriPath Imaging, Burlington, NC, USA) coletadas de mulheres da região do Vale do Ribeira, durante julho de 2013 e setembro de 2015 com citologia anterior classificada como células escamosas atípicas de significado indeterminado (ASC-US), lesão intraepitelial de baixo grau (LSIL), células escamosas atípicas, não podendo excluir lesão de alto grau (ASC-H) e células glandulares atípicas (AGC). O exame histopatológico foi realizado em 179 mulheres. As avaliações de desempenho foram calculadas usando o teste de intervalo de confiança (IC) “exato” de Clopper-Pearson de 95% pelo software MEDCALC (Medcalc Software Ltd, Ostend, Bélgica).

**Resultados** A frequência de ≥ NIC 2 foi 11,7% (21/179). A CH2 para o HPVar e a citologia de repetição para a detecção ≥ NIC 2 obteve, respectivamente, sensibilidade de 90.5% (IC 95% = 69,6–98,8) e 90,5% (IC 95% = 69,6–98,8), especificidade de 65,8% (IC 95% = 57,9–73,2) e 43,7%, (IC 95% = 35,8–51,8), valor preditivo positivo de 26,0% (IC 95% = 21,4–31,3) e 17,6%, (IC95% = 14,9–20,6), e valor preditivo negativo de 98,1% (IC 95% = 93,3–99,5) e 97,2%, (IC 95% = 90,1–99,2).

**Conclusão** No geral, a CH2 para HPVar aprimora o desempenho para detecção de ≥ NIC 2, sem comprometer a sensibilidade e proporciona maior margem de segurança do retorno ao rastreio trienal de mulheres com anormalidades citológicas prévias, sem ≥ NIC 2 subjacente.

## Introduction

Cervical cancer is the third most frequent neoplasia in the Brazilian female population, with an estimated incidence of 16,370 cases for 2018.^1^ The persistent infection of the genital tract with high-risk human papillomavirus (hrHPV) types is one of the main causes for the occurrence of this disease, which has resulted in the development of tests for HPV nucleic acid detection.^2^ ^3^ Most industrialized countries have introduced hrHPV assays; however, this methodology is not available to the women assisted by the Brazilian Unified Health System (SUS, in the Portuguese acronym), which recommend Papanicolaou (Pap) test to detect cervical cancer precursors, and the follow-up management of the women varies according to age and the type of lesion.^4^ ^5^

Women with atypical squamous cells of undetermined significance (ASC-US) and low-grade squamous intraepithelial lesion (LSIL) must repeat the test after 6 months to 3 years, depending on the age, and they are referred to colposcopy in case of persistent cytological abnormalities.^5^ However, repeat cytology in these cases may increase the anxiety of the women and delay detection of more severe lesions masked by low-grade phenotype due to its low representativeness.^6^ Cytologies classified as atypical squamous cells cannot exclude high-grade squamous intraepithelial lesion (ASC-H), high-grade squamous intraepithelial lesion (HSIL) and atypical glandular cells (AGC), which, in Brazil, are referred to immediate colposcopy.^5^ Indeed, there is a consensus that all women with HSIL require colposcopy assessment; however, there are still divergences regarding the management of women with ASC-H and AGC cytology.^6^ ^7^ Several studies have already shown that the hrHPV testing has higher sensitivity than cytology to detect cervical intraepithelial neoplasia grade 2 or worse (≥ CIN 2), besides its higher reproducibility, compared with the Pap test. Also, HPV-based cervical screening provides greater protection against invasive cancer than cytological-based screening.^8^ ^9^ ^10^ ^11^ ^12^ Nevertheless, the age must be considered when interpreting hrHPV testing results, adolescent and young women show significantly higher rates of HPV infection than women aged ≥ 30 years old.^13^

Socioeconomic, geographical, social and cultural factors seem to contribute to the lower participation of women who belong to ethnical minorities on screening programs.^14^ Atypical squamous cells of undetermined significance, LSIL, ASC-H and AGC management increases the demand for colposcopy; however, most of these abnormalities have a benign origin or regress spontaneously. We hypothesized that hrHPV testing may improve the closure of these abnormal cytologies in the context of public health, especially in the SUS. The present study aimed to evaluate the performance of the hybrid capture 2 (HC2) technique for hrHPV and cytological exam in women with previous cytology classified as ASC-US, LSIL, ASC-H, and AGC to detect ≥ CIN 2.

## Methods

### Casuistry

**Fig. 1:**
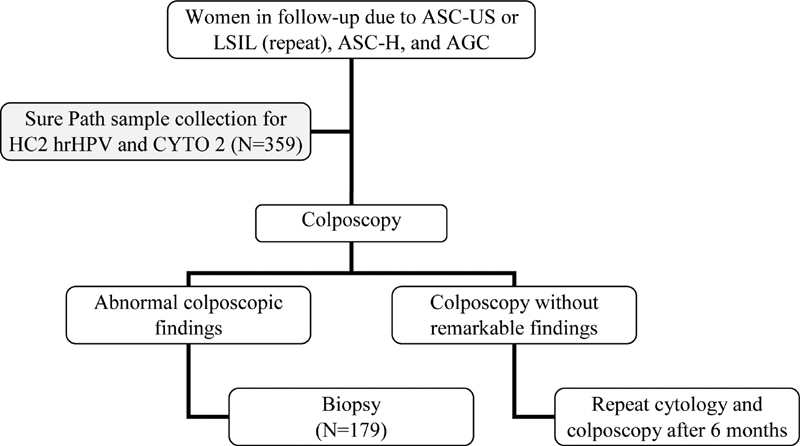
Simplified diagram of women follow-up due to ASC-US, LSIL, ASC-H, and AGC, with the insertion point of the present research.

This is a cross-sectional study conducted at the Adolfo Lutz Institute, the central public health laboratory of the state of São Paulo, Brazil, with convenience samples of women undergoing routine cervical screening between July 2013 and September 2015. The participants of the present study are residents of Vale do Ribeira, a region in the state of São Paulo, which has a part of the population living under highly vulnerable conditions, considering that 25.91% is rural population and 7.65% live under extreme poverty conditions.^15^ ^16^ The samples were referred from the Leopoldo Bevilacqua Regional Hospital or from the Vale do Ribeira Region Basic Health Units due to previous cytology classified as ASC-US, LSIL, ASC-H, and AGC, as shown in Fig. 1.

Out of the total of 359 women who were cotested using SurePath (Becton Dickinson, TriPath Imaging, Burlington, NC, USA) liquid-based cytology samples, 183 women had uterine cervix biopsy collected in the same period of the cotest, 4 of them with inconclusive histopathological results, which were excluded from the casuistry. The participants signed the free informed consent, and the present study was approved by the Research Ethics Committee of the Instituto Adolfo Lutz (CAAE: 26042213.1.0000.0059).

### Repeat Cytology (CYTO 2)

SurePath liquid-based samples were collected, with a cervical brush (Rovers Medical Devices, Lekstraat 10, NL-5347, KV Oss, The Netherlands). The liquid-based cytology (LBC) was processed and stained in an automated manner, according to the manufacturer's instructions. Cytology evaluation was conducted by researchers and a pathologist with at least 10 years of experience in cytopathology following the Brazilian nomenclature for cytopathological terminology for reporting Pap test results.^5^

### Hybrid Capture 2

Human papillomavirus was analyzed by HC2 assay (Qiagen, Gaithersburg, Maryland, EUA) for group B viruses (16, 18, 31, 33, 35, 39, 48, 51, 52, 56, 58, 59 and 68) using the residual LBC specimen, according to the instructions of the manufacturer. The viral load was determined through the quantification of light emission and expressed as a relation of the relative light unit (RLU) with the positive control (PCB), RLU/PCB. Results < 1.0 were considered negative (hrHPV-), and results ≥ 1.0 were considered positive (hrHPV + ).

### Histopathological Exam

We analyzed 179 uterine cervix fragments, fixed in formalin, histologically processed, included in paraffin, submitted to 3-µm cuts and stained with hematoxylin and eosin. Microscopical analysis was made by one single pathologist with > 30 years of experience and was classified as cervicitis, cervical intraepithelial neoplasia grade 1, 2 or 3, and squamous cell carcinoma.

### Statistical Analysis

The analyses were stratified by overall; aged groups: women < 30 years old and women ≥ 30 years old; and according to the previous cytology abnormalities: ASC-US, LSIL, ASC-H, and AGC. The cutoff for HC2 for hrHPV was relative light units (RLU) ≥ 1 pg/mL and for Repeat Cytology (CYTO 2) it was ASC-US.

We calculated the frequency of HC2 for hrHPV+ and CYTO 2 positive (CYTO 2 + ). The statistical differences between HC2 for hrHPV+ and CYTO 2+ frequencies were assessed by Exact confidence intervals, computed by the method of Clopper and Pearson using the GraphPad Quickcalcs Software program (GraphPad, La Jolla, CA, USA)

Histology was considered the gold standard method for the diagnosis of CIN. For performance evaluation of the HC2 for hrHPV and CYTO 2, women with cervicitis and CIN 1 were classified as absence of disease. Women with CIN 2, CIN 3, and squamous cell carcinoma (≥ CIN 2) were classified as presence of disease. There was no adenocarcinoma in the samples studied.

The performance evaluations were calculated using MEDCALC (Medcalc Software Ltd, Ostend, Belgium)^17^ easy-to-use statistical software (https://www.medcalc.org/calc/diagnostic_test.php). Confidence intervals (CIs) of 95% for sensitivity, specificity and accuracy are “exact” Clopper-Pearson CIs, and for the positive predictive value (PPV) and negative predictive value (NPV) are standard logit confidence intervals given by Mercaldo et al.^18^ Table 1 shows the criteria for defining positive and negative cases for statistical analysis, and Table 2 shows the definitions of the sensitivity, specificity, PPV, NPV, and accuracy.

**Table 1:** Criteria for classifying cases as true positive, false positive, true negative and false negative, based on results of HC2 for hrHPV and CYTO 2

	HC2 for hrHPV	CYTO 2
True positive	hrHPV positive with ≥ CIN 2	CYTO 2 positive with ≥ CIN 2
False positive	hrHPV positive without ≥ CIN 2	CYTO 2 positive without ≥ CIN 2
True negative	hrHPV negative without ≥ CIN 2	CYTO 2 negative without ≥ CIN 2
False negative	hrHPV negative wtih ≥ CIN 2	CYTO 2 negative with ≥ CIN 2

Abbreviations: CIN 2: cervical intraepithelial neoplasia grade 2; CYTO 2, repeat cytology; HC 2, hybrid capture 2; hrHPV; high-risk human papillomavirus.

**Table 2:** Formulas for test performance

Sensitivity	True Positive/(False Negative + True Positive)
Specificity	True Negative/(False Positive + True Negative)
PPV	sensitivity. prevalence/[sensitivity. prevalence + (1-specificity). (1-prevalence)]
NPV	specificity. (1-prevalence)/[(1-sensitivity). prevalence + specificity. (1-prevalence)]
Accuracy	sensitivity. prevalence + Specificity. (1-prevalence)

Abbreviations: NPV, negative predictive value; PPV, positive predictive value.

## Results

The HC2 test for hrHPV and cytology was conducted on liquid-based material in 359 women. The mean age of the participating women was 39.8 years old (15 to 79 years old), with mean ages of 37.0 years old for women with hrHPV + , 39.1 years old for women with CYTO 2 + , 41.1 years old for women with hrHPV-, and 40.9 years old for women with CYTO 2-.

### Distribution and Percentage of HC2 for hrHPV and CYTO 2 Positivity

The hrHPV+ and CYTO 2+ absolute and relative frequencies, their respective 95%CIs, and the total of analyzed samples are described in Table 3, stratified by overall; age groups (women < 30 years old and women aged ≥ 30 years old); and previous cytologic abnormalities (ASC-US, LSIL, ASC-H, and AGC). Overall, the hrHPV+ rate was lower than the CYTO 2+ rate. Women aged ≥ 30 years old showed a lower hrHPV+ rate than CYTO 2+ rate. The hrHPV+ rate in women < 30 years old was significantly higher than in women aged ≥ 30 years old; however, there was no significant difference between CYTO 2+ rate in women < 30 years old and in women aged ≥ 30 years old. According to the previous cytologic abnormalities stratification, we did not observe a statistical difference between hrHPV+ and CYTO 2+ frequencies (Table 3).

**Table 3:** Distribution and percentage of HC2 for hrHPV+ and CYTO 2+ stratified by overall, age groups and previous cytological abnormalities

	hrHPV+ (%)	95%CI	CYTO 2+ (%)	95%CI	Total
Overall	113 (31.5)	0.27–0.37	170 (47.4)	0.42–0.53	359
< 30 years old	45 (45.5)	0.35–0.56	51 (51.5)	0.41–0.62	99
≥ 30 years old	68 (26.2)	0.21–0.32	119 (45.8)	0.40–0.52	260
Previous cytology abnormalities					
ASC-US	25 (20.5)	0.14–0.29	44 (36.1)	0.28–0.45	122
LSIL	54 (38.6)	0.30–0.47	68 (48.6)	0.40–0.57	140
ASC-H	30 (43.5)	0.32–0.56	45 (65.2)	0.53–0.76	69
AGC	4 (14.3)	0.04–0.33	13 (46.4)	0.28–0.66	28

Abbreviations: AGC, atypical glandular cells; ASC-H, atypical squamous cells, cannot exclude high-grade squamous intraepithelial lesions; ASC-US, atypical squamous cells of undetermined significance; CYTO2 + , repeat cytology atypical squamous cells of undetermined significance or worse; hrHPV + , hybrid capture 2 for high-risk human papillomavirus (RLU > 1pg/mL); LSIL, low-grade squamous intraepithelial lesion.

### Performance of HC2 for hrHPV and CYTO 2

The performance of HC2 for hrHPV and CYTO 2 was calculated based on the biopsy results. Table 4 shows the distribution and prevalence of ≥ CIN 2 in a total of women with the histopathological exam. The HC2 for hrHPV performance is shown in Table 5, and CYTO 2 performance is shown in Table 6, both assessed in the population with biopsy (Table 4).

**Table 4:** Distribution and prevalence of ≥ CIN 2 stratified by overall, age groups and previous cytologic abnormalities

	≥ CIN 2	Prevalence	Total
Overall	21	11.7	179
Age groups			
< 30 years old	7	14.0	50
≥ 30 years old	14	10.9	129
Previous cytology abnormalities			
ASC-US	3	7.1	43
LSIL	5	8.2	61
ASC-H	11	18.3	60
AGC	2	12.5	16

Abbreviations: AGC, atypical glandular cells; ASC-H, atypical squamous cells, cannot exclude high-grade squamous intraepithelial lesions; ASC-US, atypical squamous cells of undetermined significance; CIN 2: cervical intraepithelial neoplasia grade 2.

**Table 5:** HC2 for hrHPV performance stratified by overall, age groups and previous cytology abnormalities

	n	TP	FP	FN	TN	hrHPV + (%)	Sensitivity (95% CI)	Specificity (95% CI)	PPV (95% CI)	NPV (95% CI)	Accuracy (95% CI)
Overall (age 15–79 years old)	179	19	54	2	104	40.8	90.5 (69.6–98.8)	65.8 (57.9–73.2)	26.0 (21.4–31.3)	98.1 (93.3–99.5)	68.7 (61.4–75.4)
Age groups											
< 30 years old	50	6	26	1	17	64.0	85.7 (42.1–99.6)	39.5 (25.0–56.0)	18.8 (13.6–25.4)	94.4 (72.7–99.1)	46.0 (31.8–60.7)
≥ 30 years old	129	13	28	1	87	31.8	92.9 (66.1–99.8)	75.7 (66.8–83.2)	31.7 (24.6–39.8)	98.9 (92.9–99.8)	77.5 (69.3–84.4)
Previous cytology abnormalities											
ASC-US/LSIL	103	8	36	–	59	38.2	100 (63.1–100.0)	62.1 (51.6–71.9)	18.2 (14.7–22.3)	100 -	65.1 (55.0–74.2)
ASC-H/AGC	76	11	18	2	45	42.7	84.6 (54.5–98.1)	71.4 (58.7–82.1)	37.9 (28.0–49.0)	95.7 (86.2–98.8)	73.7 (62.3–83.1)

Abbreviations: AGC, atypical glandular cells; ASC-H, atypical squamous cells, cannot exclude high-grade squamous intraepithelial lesions; ASC-US, atypical squamous cells of undetermined significance; FN, false negatives (HPV negative, ≥ CIN 2); FP, false positives (HPV positive, ≤ CIN 1); LSIL, low-grade squamous intraepithelial lesion; NPV, negative predictive value; PPV, positive predictive value; TN, true negatives (HPV negatives, ≤ CIN 1); TP; true positives (HPV positive ≥ CIN 2).

Adapted from Cotton et al. (2010).^21^

**Table 6:** CYTO 2 performance stratified by the result of previous cytology results

	n	TP	FP	FN	TN	Cyto 2+ (%)	Sensitivity (95% CI)	Specificity (95% CI)	PPV (95% CI)	NPV (95% CI)	Accuracy (95% CI)
**Overall** **(age 15–79 years old)**	179	19	89	2	69	60.3	90.5 (69.6–98.8)	43.7 (35.8–51.8)	17.6 (14.9–20.6)	97.2 (90.1–99.2)	49.2 (41.6–56.7)
**Age groups**											
**<** **30 years old**	50	6	27	1	16	66.0	85.7 (42.1–99.6)	37.2 (23.0–53.3)	18.2 (13.2–24.5)	94.1 (71.4–99.0)	44.0 (30.0–58.8)
**≥** **30 years old**	129	13	62	1	53	58.1	92.9 (66.1–99.8)	46.1 (36.8–55.6)	17.3 (14.4–20.8)	98.2 (88.8–99.7)	51.2 (42.2–60.1)
**Previous cytology abnormalities**											
**ASC-US/LSIL**	103	8	49	–	46	54.4	100 (63.1–100.0)	48.4 (38.0–58.9)	14.0 (11.8–16.6)	100.0	52.4 (42.4–62.4)
**ASC-H/AGC**	76	11	41	2	22	68.4	84.6 (54.6–98.1)	34.9 (23.2–48.0)	21.2 (16.7–26.5)	91.7 (74.7–97.6)	43.4 (32.1–55.3)

Abbreviations: AGC, atypical glandular cells; ASC-H, atypical squamous cells, cannot exclude high-grade squamous intraepithelial lesions; ASC-US, atypical squamous cells of undetermined significance; FN, false negatives (CYTO 2 negative, ≥ CIN 2); FP, false positives (CYTO 2, ≤ CIN 1); LSIL, low-grade squamous intraepithelial lesion; NPV, negative predictive value; PPV, positive predictive value; TN, true negatives (CYTO 2 negatives, ≤ CIN 1); TP; true positives (CYTO 2 positive ≥ CIN 2).

Adapted from Cotton et al. (2010).^21^

The specificity and accuracy of the HC2 for hrHPV in women aged ≥ 30 years old were higher than in women < 30 years old. There was no statistical difference between the performance of HC2 for hrHPV in women with previous ASC-US/LSIL and ASC-H/AGC (Table 5).

There was no statistical difference between the performance of CYTO 2 in women < 30 years old and in women ≥ 30 years old, and between previous ASC-US/LSIL and ASC-H/AGC (Table 6).

Overall, the HC2 for hrHPV showed significantly higher performance than CYTO 2 for the detection of ≥ CIN 2 in the following categories: specificity (65.8%, 95%CI = 57.9–73.2, and 43.7%, 95%CI = 35.8–51.8, respectively), PPV (26.0%, 95%CI = 21.4–31.3, and 17.6%, 95%CI = 14.9–20.6, respectively), and accuracy (68.7%, 95%CI = 61.4–75.4, and 49.2%, 95%CI = 41.6–56.7, respectively). The HC2 for hrHPV in women ≥ 30 years old showed a significantly higher performance than CYTO 2 for the detection of ≥ CIN 2 regarding specificity (75.7%, 95%CI = 66.8–83.2, and 46.1%, 95%CI = 36.8–55.6, respectively), PPV (31.7%, 95%CI = 24.6–39.8, and 17.3%, 95%CI = 14.4–20.8, respectively), and accuracy (77.5%, 95%CI = 69.3–84.4, and 51.2%, 95%CI = 42.2–60.1, respectively). There was no statistic difference between HC2 for hrHPV and CYTO 2 performance in women < 30 years old. Women with previous ASC-H/AGC showed a significant higher performance of HC2 for hrHPV than CYTO 2 for the detection of ≥ CIN 2 regarding specificity (71.4%, 95%CI = 58.7–82.1, and 34.9%, 95%CI = 23.2–48.0, respectively), PPV (37.9%, 95%CI = 28.0–49.0, and 21.2%, 95%CI = 16.7–26.5, respectively), and accuracy (73.7%, 95%CI = 62.2–83.1, and 43.4%, 95%CI = 32.1–55.3, respectively). There was no statistic difference between HC2 for hrHPV and CYTO 2 in women with ASC-US/LSIL (Tables 5 and 6).

## Discussion

The greater specificity, PPV and accuracy for ≥ CIN 2 detection observed in HC2 for hrHPV assay concerning repeat cytology assay observed in the present study performed in women from the Vale do Ribeira region with previous cytology abnormalities suggest that the biomolecular method could improve the triage for the colposcopic referral, especially in women aged ≥ 30 years old.

According to meta-analyses that assessed LBC and HC2 for the detection of ≥ CIN 2 in cross-sectional studies from Africa, Asia, North America, Oceania, Pacific, Central, and South America, the performance of LBC at the threshold of ASC-US or worse, with median sample size of 3,843, the sensitivities of the tests ranged from 52 to 94%, and specificities ranged from 52 to 98%. For HC2, the median sample size was 4,195, and the sensitivities of the tests ranged from 61 to 100%, and specificities ranged from 64 to 95%.^19^ Compared with this study, our results showed similar values regarding HC2 sensitivity and specificity; however, the repeat cytology specificity was lower than those in the meta-analyses. Nevertheless, in the 10 articles included in the meta-analysis by Arbyn et al^20^ that evaluated the performance of the detection of ≥ CIN 2 by repeat cytology in samples with previous ASC-US, considering ASC-US as a cutoff, one showed a specificity of 45%, a rate similar to that observed in our study. On the other hand, the same study showed 68.4% of absolute specificity in repeat cytology, 60.7% in HC2, 71.5% of absolute sensitivity in repeat cytology, and 90.9% in HC2.^20^ Furthermore, the results of sensitivity, specificity, and PPV showed considerable variation in the literature for both tests.^19^ ^20^ ^21^

Women with ASC-US and LSIL abnormalities have a higher risk for underlying ≥ CIN 2 than women with normal cytology, and there has been controversy about the better management of the women with minor cytological abnormalities.^22^ ^23^ ^24^ In our study, we did not observe greater HC2 for hrHPV performance in women with previous ASC-US/LSIL than repeat cytology. Low-grade squamous intraepithelial lesion should be considered only as a transitory expression of the HPV infection due to its high rate of spontaneous regression, especially in young women.^25^ ^26^ In women < 30 years old, ASC-US and LSIL are common and, although they may indicate an underlying ≥ CIN 2, the spontaneous regression potential is high.^27^ Younger women, in comparison to older women, are more likely to have false-positive cytology and less likely to be tested positive for the hrHPV testing.^28^

Therefore, HC2 for hrHPV could improve the triage for colposcopy mainly in women aged ≥ 30 years old, the specificity for this age group was significantly higher than repeat cytology and HC2 for hrHPV in women < 30 years old. In Cotton et al,^21^ the multicenter individually randomized controlled trial (*n* = 4,439) showed 63.9% specificity in HPV test for ≥ CIN 2 detection in women aged between 20 and 59 years old with recruitment based on morphological findings as mild dyskaryosis and borderline nuclear abnormalities, similar to our study. Nevertheless, in the same study, the specificity increases along with the age group, with higher specificity in older women.^21^

According to the meta-analysis by Arbyn et al,^20^ the triage of women with a cytological test result of ASC-US using the HC2 test is more sensitive, and equally specific to repeat cytology to detect underlying ≥ CIN2. The triage of women with a cytological test result of LSIL through the HC2 test is more sensitive but substantially less specific than repeat cytology to detect ≥ CIN2. The specificity of HC2 improves for older women.^20^ On the other hand, its lower specificity could have cost implications because of the referral of a large number of women with false-positive results to colposcopy, and the PPV should be considered for colposcopy referral.^19^ ^20^ In our study, we observed a similar sensitivity between HC2 for hrHPV and repeated cytology, but greater specificity and PPV in the hrHPV test, especially in women ≥ 30 years old. However, we had some limitations, as a small casuistry and analysis from women of a specific geographic region, which could affect the predictive value. Therefore, in practice, for the introduction of HPV assay in the Brazilian public health system, cost-effectiveness studies with a larger and more diversified casuistry will be necessary.

The cytological abnormalities classified as ASC-H are frequently associated with high positivity rates for hrHPV, with a relatively high risk of ≥ CIN 2, ranging from 13 to 66%.^7^ However, these abnormalities are also associated with benign conditions, such as squamous metaplasia with degenerative characteristics, atrophy, reserve cell hyperplasia, hormonal effect, among others.^29^ Our study detected hrHPV in 43.5% of the ASC-H, and the prevalence of ≥ CIN 2 was 18.3%. A Brazilian study with women assisted at private clinics reported positivity for hrHPV testing in 67.7% (40/68) and 50% (17/34) prevalence of ≥ CIN2 in women with ASC-H.^30^ Although the hrHPV test may assist in the differentiation between benign conditions and pre-neoplastic lesions, false-negative for ≥ CIN 2 may also occur. In the present study, HC2 was positive in 81.8% of the ASC-H with histopathological ≥ CIN 2. A study made in Belgium showed that 3% (1/32) of the ASC-H associated with ≥ CIN 2 tested negative on HC2 for hrHPV.^31^

For the AGC category, a substantially higher association is observed for CIN than the ASC-US and LSIL categories.^32^ Some studies suggest that AGC may precede malignant cervical or endometrial neoplasia and that it is associated with an increased risk of ≥ CIN 2 and of underlying in situ or invasive adenocarcinoma.^33^ ^34^ On the other hand, these abnormalities can be associated with benign conditions, such as tubal metaplasia, tubular hyperplasia or polyps.^35^ One study from Ireland showed that out of 146 women referred to colposcopy following a single AGC with histologic results, 30 (20.5%) were diagnosed with ≥ CIN 2 (CIN 2, CIN 3, adenocarcinoma and cancer).^36^ In our study, the ≥ CIN 2 prevalence for the women submitted to biopsy due to AGC was 12.5%.

In Brazil, women with ASC-H and AGC are referred immediately to colposcopy. Women with biopsies without ≥ CIN 2 are kept on a semi-annual follow-up with cytology and colposcopy until the exclusion of invasive disease.^5^ In the present study, HC2 for hrHPV had greater performance in women with previous ASC-H/AGC than repeat cytology. Therefore, women with persistent ASC-H or AGC without significant colposcopy could benefit from the result of the hrHPV test, since its positivity may indicate the need to investigate the endocervical canal and hrHPV negatives samples reinforce the hypothesis that these cytological abnormalities are probably not associated to ≥ CIN 2.^5^ Thus, the work overload may be minimized on medium-complexity units, as well as the stress and anxiety of women with these types of cytological results.

Some aspects may have caused bias in our results. Women were referred for colposcopy based on previous cytology abnormalities, which avoided the possibility of management-based on hrHPV positivity influencing on the ascertainment of ≥ CIN 2. Also, we did not obtain the colposcopy results of women without biopsy, neither information about the evaluation of the transformation zone of these women. The participants of the present study were in follow-up due to mild and borderline abnormalities, and women with HSIL or worse in cytology results were not included.

The strength of our study is that it is a routine extracted from the Brazilian SUS reality, without changes in the recommended guidelines. Moreover, it included women from the Vale do Ribeira, a low human development index region, with limited access to biomolecular tests.

## Conclusion

Overall, HC2 for hrHPV improved performance in the detection of ≥ CIN 2, without compromising sensitivity concerning the repeat cytology, being greater for women ≥ 30 years old. In women with previous ASC-US/LSIL, hrHPV testing had the same impact as the repeat cytology. In previous ASC-H/AGC, the hrHPV test had greater performance. Therefore, HC2 for hrHPV could be useful in the colposcopy triage of women with previous abnormalities, especially in women ≥ 30 years old, and the negative result of HC2 for hrHPV in women with ASC-H/AGC, without significant lesions confirmed by histopathology, makes it safer for them to return to the triennial screening protocol.
